# ^18^FDG PET/CT in Pulmonary Carcinosarcoma and Brain Metastasis

**DOI:** 10.22038/AOJNMB.2019.13360

**Published:** 2019

**Authors:** Hamideh Abbasain, Ramin Sadeghi, Farshad Emami, Vahid Reza Dabbagh Kakhki

**Affiliations:** 1 Nuclear Medicine Research Center, Mashhad University of Medical Sciences, Mashhad, Iran; 2 Nuclear Medicine and , Department of Molecular Imaging, Imam Reza International University, Mashhad, Iran

**Keywords:** Brain metastasis, Carcinosarcoma, FDG PET, Lung cancer

## Abstract

Carcinosarcoma is a rare type of cancer that is composed of a mixture of sarcomatous and carcinomatous elements. Pulmonary carcinosarcoma has a 25% five-year survival rate with a prognosis poorer than other non-small cell lung carcinomas. Herein, we report a case of pulmonary carcinosarcoma and its ^18^F-FDG PET/CT findings. A 61-year-old male patient presented with brain symptoms, including headache, nausea, right hemiplegia, and few attacks of seizures. He underwent brain computed tomography (CT) scan showing a brain lesion in the left parietal lobe. The patient underwent excisional biopsy, and brain lesion was removed. The results of tissue sampling were indicative of carcinosarcoma. Based on anatomical imaging and evidence of pulmonary lesion, the patient underwent ^18^FDG PET/CT that revealed a heterogeneous mass on the upper lobe of the left lung. An intense FDG uptake was observed along the rim of the mass; however, no FDG uptake was observed in the center of the mass. There were multiple mediastinal lymph nodes with a high FDG uptake. Pulmonary carcinosarcoma was confirmed by tissue sampling.

## Introduction

Carcinosarcoma is a rare type of cancer that is composed of a simultaneous mixture of sarcomatous (mesenchymal malignancy) and carcinomatous (epithelial malignancy) elements in a single tumor (1, 2). Pulmonary carcinosarcoma accounts for less than 1% of all lung cancers (3). Accordingly, only a few cases have been reported in the literature (1-6). Pulmonary carcinosarcomas are a heterogeneous group of non-small cell lung carcinomas that have a sarcoma-like component (6). They have a 25% five-year survival rate with a prognosis poorer than other non-small cell lung carcinomas (6). ^18^FDG positron emission tomography/computed tomography (^18^FDG-PET/CT) is widely used for the detection of different malignancies. Few studies have reported the use of ^18^FDG-PET/CT imaging for pulmonary carcinosarcoma cases. However, there is no report regarding the ^18^FDG-PET/CT imaging of pulmonary carcinosarcoma with brain metastases in the literature. 

## Case report

**Figure 1:**
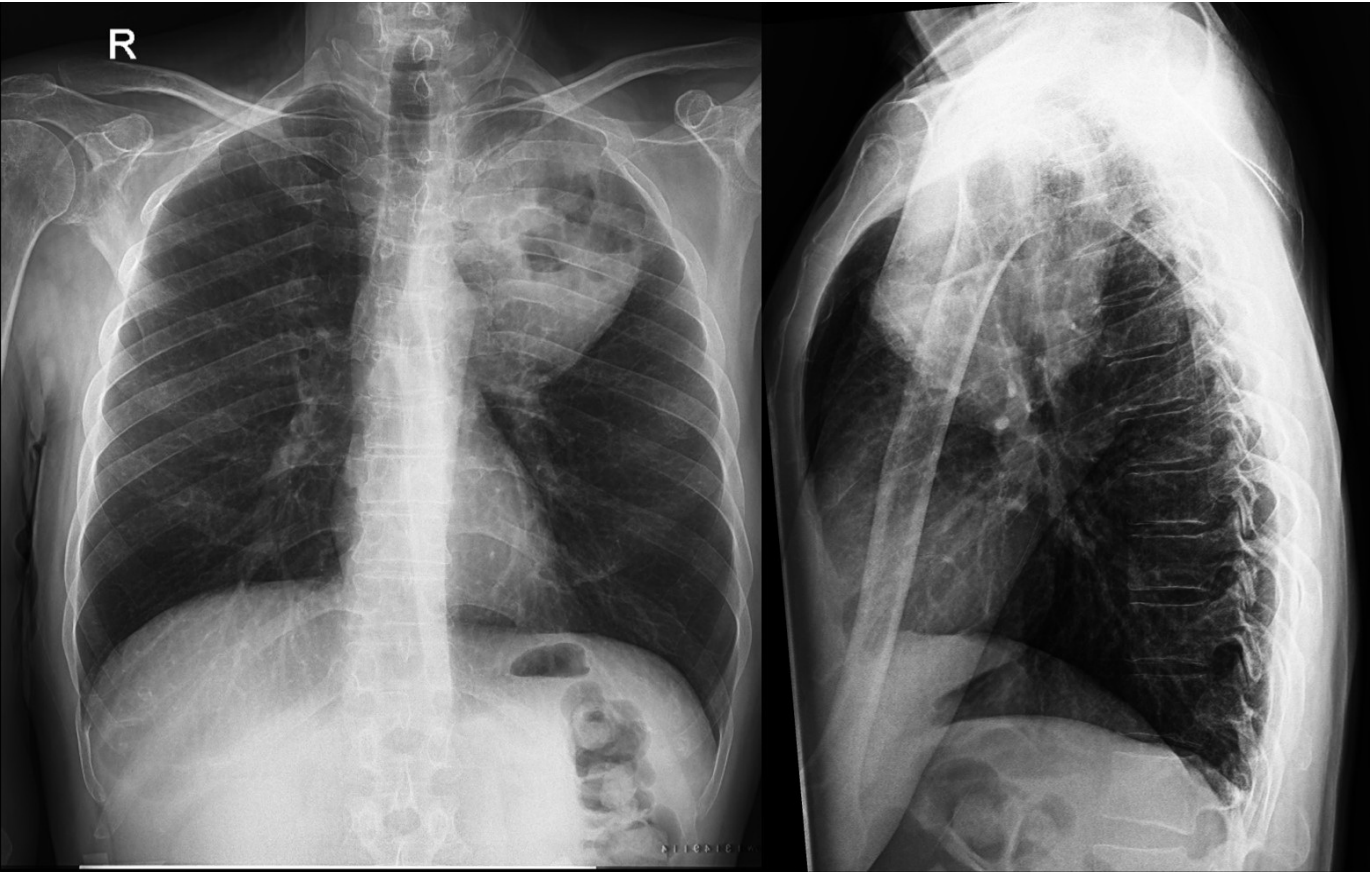
Brain CT scan showing the brain resected lesion in the left parietal lobe

**Figure 2:**
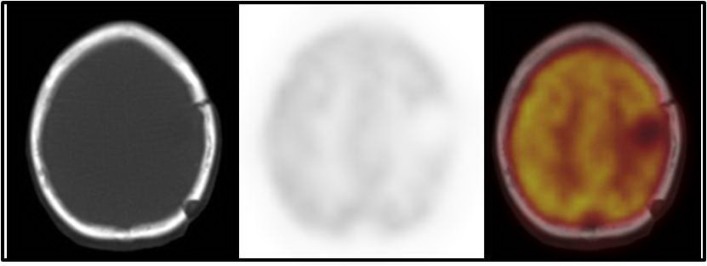
Chest radiography showing a large mass lesion in the upper lobe of the left lung

The patient was a 61-year-old male who complained of headache, nausea, right hemiplegia, and few attacks of seizures. He underwent brain computed tomography (CT) scan showing a brain lesion in the left parietal lobe. The patient underwent excisional biopsy, and brain lesion was removed (Figure 1). The results of tissue sampling were indicative of carcinosarcoma. He had mild dyspnea and cough. Because of detecting a pulmonary lesion in the chest radiography (Figure 2), the patient was subjected to ^18^FDG-PET/CT. 

## Results

**Figure 3:**
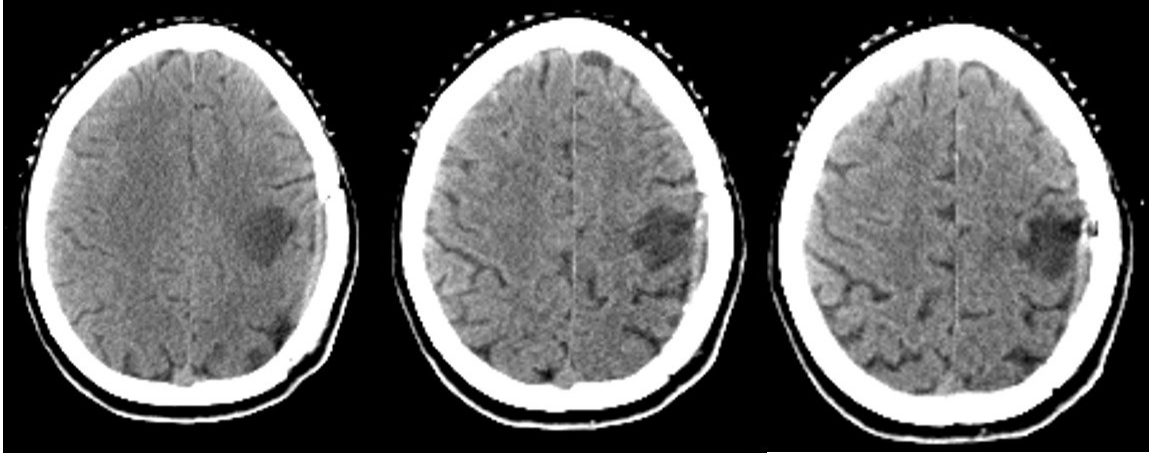
^18^FDG-PET/CT showing no FDG uptake in the region of the resected brain mass

**Figure 4:**
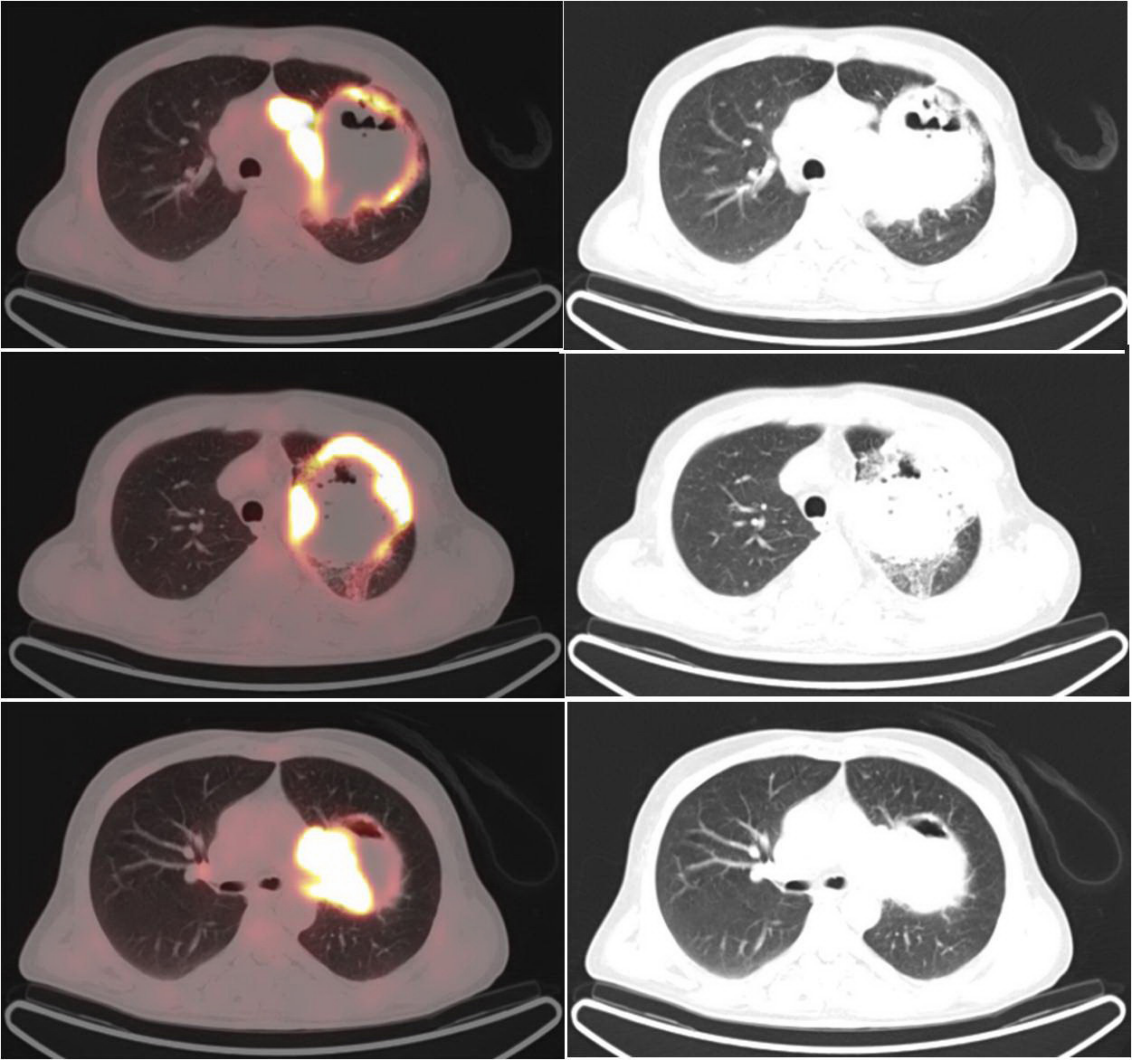
^18^FDG-PET/CT showing a heterogeneous mass on the left upper lobe surrounded with a rim of intense FDG uptake (SUV_max_=10.8) (There was no FDG uptake in the center of the mass. There were multiple mediastinal lymph nodes with a high FDG uptake in the aortic region with a SUV_max_ of 15.3.)

**Figure 5:**
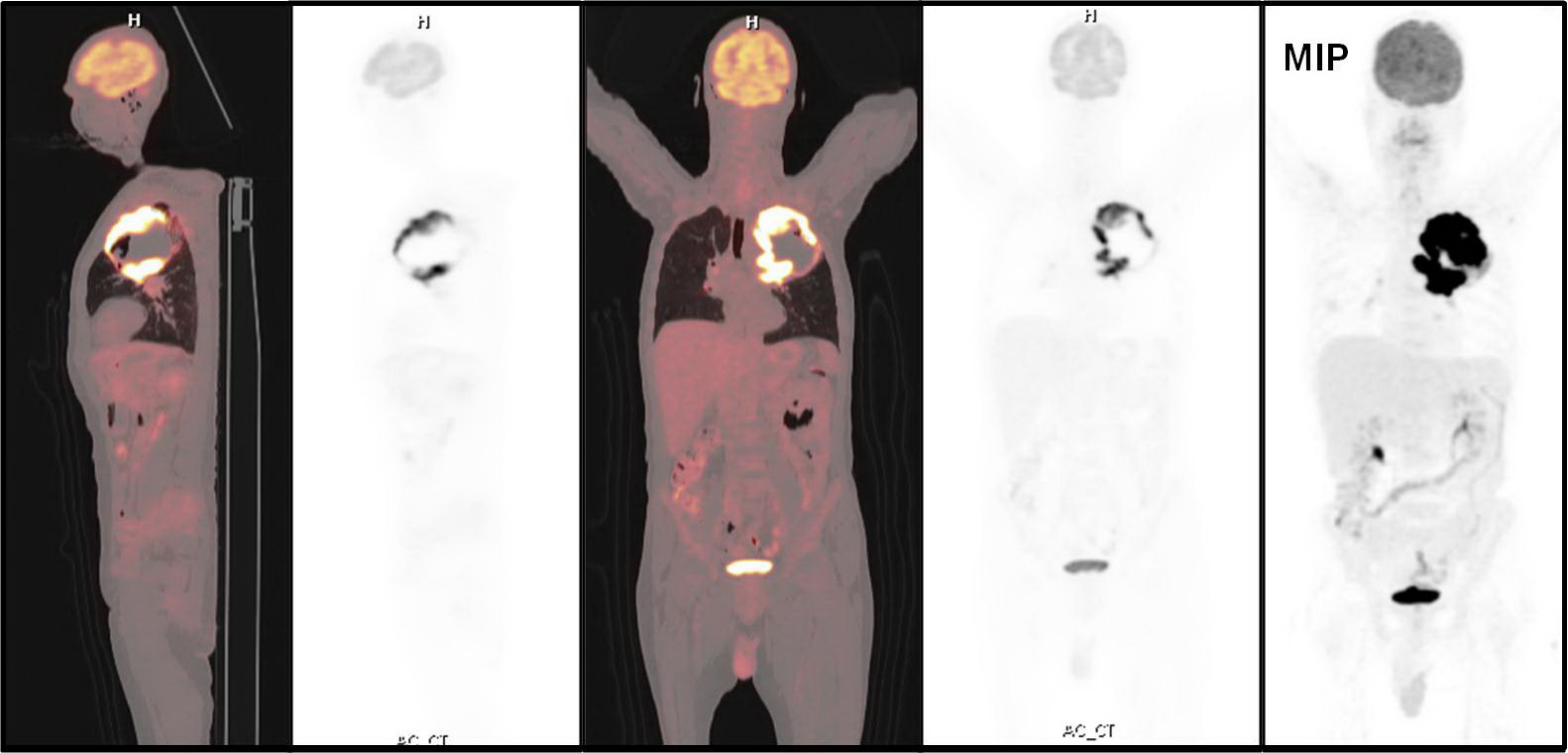
Maximum projection and sagittal image of FDG PET/CT showing a large mass on the upper lobe of the left lung with a rim of high FDG uptake and mediastinal lymph node metastases

The^18^FDG-PET/CT revealed a large heterogeneous lesion in the left lung (figures 3, 4, and 5). In the left lung, a heterogeneous mass was seen on the left upper lobe, surrounded by a rim of intense FDG uptake (figures 4 and 5). Lack of uptake in the center of the left lung lesion may be due to central necrosis. Maximum standardized uptake value (SUV_max_) was 10.8. There were extensive and multiple mediastinal lymph nodes with a high FDG uptake in the aortic, left prevascular, and left hilar regions (SUV_max_=15.3). Biopsy of the left lung lesion revealed the sarcomatoid carcinoma of the lung. 

## Discussion

In this report, we discussed an unusual presentation of pulmonary carcinosarcoma with brain metastasis. The PET/CT is used for diagnosis, primary staging, detection of the primary source, identification of a suitable site for biopsy, surveillance, therapy response evaluation, and detection of recurrence in many cancers (7). The PET/CT can be useful in the detection of tumor location, as well as the determination of disease prognosis using standardized uptake values (7, 8). The FDG-PET/CT is also helpful to determine the unknown primary tumor sites for metastatic lesions. 

The Medline and SCOPUS search revealed a few cases of pulmonary carcinosarcoma with brain metastasis (9-11). In our patient, there was a rim of intense FDG uptake around the pulmonary lesion, with no uptake in the center, which may be due to central necrosis. There was an intense tracer uptake in the metastatic mediastinal lymph nodes.

In many patients with a brain metastasis of unknown primary origin, neurological symptoms are the first clinical manifestations of malignancy. The lung is the most primary tumor site responsible for these patients. The whole body FDG-PET/CT imaging is useful for the detection of the primary site of the metastases of unknown origin (12). This modality is commonly used for the staging of lung cancer. In our case, the brain lesion was resected; therefore, FDG uptake was not seen. 

However, the routine staging of the brain status in patients with lung cancer is based on brain MRI (13). The MRI is the imaging modality of choice used to evaluate the space occupying the lesions in the brain. A high physiologic accumulation of FDG in the brain has been proved to be a challenge in identifying brain metastases. Regarding this, the sensitivity of FDG PET/ CT for brain metastases may not be high (13-15). 

## References

[1] Yamazaki K A (2003 ). A gastric carcinosarcoma with neuroendocrine cell differentiation and undifferentiated spindle-shaped sarcoma component possibly progressing from the conventional tubular adenocarcinoma; an immunohistochemical and ultrastructural study. Virchows Arch.

[2] Li B, Zhang Y, Hou J, Yu H, Shi H (2016 ). Primary Liver Carcinosarcoma and 18F-FDG PET/CT. Clin Nucl Med.

[3] Braham E, Ben Rejeb H, Aouadi S, Kilani T, El Mezni F (2014). Pulmonary carcinosarcoma with heterologous component: report of two cases with literature review. Ann Transl Med.

[4] Sakane T, Okuda K, Hattori H, Watanabe T, Oda R, Tatematsu T, Yokota K, Haneda H, Inagaki H, Nakanishi R (2018 ). Blastomatoid pulmonary carcinosarcoma: A rare case report and review of the literature. Thorac Cancer.

[5] Borczuk AC (2018 ). Uncommon Types of Lung Carcinoma With Mixed Histology: Sarcomatoid Carcinoma, Adenosquamous Carcinoma, and Mucoepidermoid Carcinoma. Arch Pathol Lab Med.

[6] Ciarallo A, Makis W, Novales-Diaz JA, Lisbona R (2012 ). Sarcomatoid carcinoma (carcinosarcoma) of the lung mimics malignant pleural mesothelioma on 18F-FDG PET/CT: a report of 2 cases. Clin Nucl Med.

[7] Li B, Zhang Y, Hou J, Cai L, Zhou J, Shi H (2015 ). Gastric Carcinosarcoma and 18F-FDG PET/CT. Clin Nucl Med.

[8] Sato Y, Shimozono T, Kawano S, Toyoda K, Onoe K, Asada Y, Hayashi T (2001 ). Gastric carcinosarcoma, coexistence of adenosquamous carcinoma and rhabdomyosarcoma: a case report. Histopathology.

[9] Mine T, Mizukami M, Tomita T, Iri H, Mihara H (1969 ). Case of lung carcinosarcoma with brain metastasis. No To Shinkei.

[10] Kanehisa Y, Kaji M (1993 ). A case of pulmonary carcinosarcoma presenting cerebellar metastasis. Rinsho Shinkeigaku.

[11] Oliveira MF, Watanabe SC, Andrade MP, Rotta JM, Pinto FC (2013 ). Sarcomatoid carcinoma of the lung with brain metastases. J Bras Pneumol.

[12] Al-Zaghal A, Raynor WY, Seraj SM, Werner TJ, Alavi A (2019 ). FDG-ET imaging to detect and characterize underlying causes of fever of unknown origin: an unavoidable path for the foreseeable future. Eur J Nucl Med Mol Imaging.

[13] Ho KC, Toh CH, Li SH, Liu CY, Yang CT, Lu YJ, Su TP, Wang CW, Yen TC (2018). Prognostic impact of combining whole-body PET/CT and brain PET/MR in patients with lungadenocarcinoma and brain metastases. Eur J Nucl Med Mol Imaging.

[14] Yamamoto AJ, Zhuang H, Alavi A (2001). Detection of cranial metastases by F-18FDG positron emission tomography. Clin Nucl Med.

[15] Coleman RE (2001 ). PET in lung cancer staging. Q J Nucl Med.

